# Adjusting team involvement: a grounded theory study of challenges in utilizing a surgical safety checklist as experienced by nurses in the operating room

**DOI:** 10.1186/1472-6955-11-16

**Published:** 2012-09-07

**Authors:** Hilde Valen Wæhle, Arvid Steinar Haugen, Eirik Søfteland, Esther Hjälmhult

**Affiliations:** 1 Department of Anesthesia and Intensive Care, Haukeland University Hospital, Bergen, Norway; 2 Department of Medicine, University of Bergen, Bergen, Norway; 3 Faculty of Health and Social Sciences, Bergen University College, Bergen, Norway

## Abstract

**Background:**

Even though the use of perioperative checklists have resulted in significant reduction in postoperative mortality and morbidity, as well as improvements of important information communication, the utilization of checklists seems to vary, and perceived barriers are likely to influence compliance. In this grounded theory study we aimed to explore the challenges and strategies of performing the WHO’s Safe Surgical Checklist as experienced by the nurses appointed as checklist coordinators.

**Methods:**

Grounded theory was used in gathering and analyzing data from observations of the checklist used in the operating room, in conjunction with single and focus group interviews. A purposeful sample of 14 nurse-anesthetists and operating room nurses as surgical team members in a tertiary teaching hospital participated in the study.

**Results:**

The nurses’ main concern regarding checklist utilization was identified as “how to obtain professional and social acceptance within the team”. The emergent grounded theory of “adjusting team involvement” consisted of three strategies; distancing, moderating and engaging team involvement. The use of these strategies explains how they resolved their challenges. Each strategy had corresponding conditions and consequences, determining checklist compliance, and how the checklist was used.

**Conclusion:**

Even though nurses seem to have a loyal attitude towards the WHO’s checklist regarding their task work, they adjusted their surgical team involvement according to practical, social and professional conditions in their work environment. This might have resulted in the incomplete use of the checklist and therefore a low compliance rate. Findings also emphasized the importance of: a) management support when implementing WHO’s Safe Surgical Checklist, and b) interprofessional education approach to local adaptation of the checklists use.

## Background

Providing healthcare is inherently interdisciplinary, involving physicians, nurses and allied health professionals from different specialties. It is widely recognized in patient safety literature that team performance is crucial in providing safe patient care [1] and that many of the factors contributing to adverse events in healthcare, originate from flawed teamwork, rather than from lack of clinical skills [2,3]. Various factors affecting the quality and safety of patient care have been identified as poor communication, poor coordination, lack of understanding of roles and shared goals for patient care between the professional groups, limited sharing of information between team members, disagreements, and aggressions [4-6]. Thus teamwork has been addressed as a key factor in system-based interventions to improve patient safety and medical education standards [4].

In 2008 the World Health Organization launched the Safe Surgical Checklist [7] and great efforts are being made on its implementation in hospitals around the world. However, there is an ongoing debate in leading medical journals as to whether a technical solution such as the checklist really can contribute to safer patient care, whilst effective teamwork per se is more crucial to providing safe patient care, and teamwork must be distinguished from task work [8]. The relationship between culture change, improved coordination and reduction of errors has been well established from research in the aviation industry [9]. However, even though a checklist might be a feasible and efficient tool that promotes operating room (OR) team cohesion and information exchange [9-11], the attitudes of personnel involved towards checklists may vary, and attitudes and experiences are likely to influence compliance [12]. The second Global Patient Safety Challenge aimed to improve surgical outcomes for all patients, and ten basic essential objectives for any surgical case were compiled into the WHO’s Surgical Safety Checklist. According to the WHO’s implementation manual, the checklist provides a tool for two purposes: enabling consistency in safety for patients, and introducing and maintaining a culture that values achieving it [7].

Implementation of the WHO’s “Safe Surgical Checklist” in all surgical units at corresponding hospitals, was initiated by the Western Norway Regional Health Authority (WNRHA) in 2009. The checklist was translated to Norwegian by the Norwegian Knowledge Centre for the Health Services and the WNRHA’s Safe Surgery study group. In 2009-2011 this Norwegian version was introduced to all members of the surgical team [nurse anesthetists, consulting anesthetists, anesthesiologists, chief- and consultants of surgery, and operating room nurses] including the heads of the different surgical departments, and implemented in all surgical units at this University Hospital. In each unit the checklist contents were revised twice, based on feedback from the users during the first three month period.

### Performing the WHO’s checklist

In accordance with the WHO implementation manual, a checklist coordinator was appointed for each of the three different parts of the checklist; the nurse anesthetist before induction of anesthesia (Sign In), and the operating room (OR) nurse before skin incision (Time Out) and before the patient leaves the operating room (Sign Out) [7]. The responsibility of coordinating the checklist utilization was thus left exclusively to the nursing profession.

### Sign in

Before induction of anesthesia, the nurse anesthetist should verbally confirm with the patient his or her identity, the surgical site and the procedure being performed. If appropriate, a visual confirmation that the operative site had been marked should follow. The nurse anesthetist should verbally review with the anesthesia professionals and the circulating nurse the patient’s risk of blood loss, airway difficulties and allergies, and also whether a safety check of the anesthesia machine and medications has been completed.

### Time out

In this phase the entire team should pause immediately before the skin incision to confirm out loud that the correct operation is being performed on the correct patient and on the correct site. All team members should then verbally review with one another, in turn, the critical elements of their plan for the operation, using the checklist questions for guidance. They should also confirm that prophylactic antibiotics have been administered within the previous 60 minutes, and that essential imaging is displayed as appropriate.

### Sign out

In the last phase, the team should review together the operation that has been performed. This includes completion of sponge and instrument counts and the labeling of any surgical specimens obtained. They should also review key plans and concerns for postoperative management and recovery before moving the patient from the operating room.

In a study by Høyland and colleagues, nurses were largely in favor of the checklist and looked upon it as a platform to improve communication and clarify needs required for the surgical procedure [13]. Furthermore, Vats et al. found that the steep hierarchy of the surgical team served as a barrier to nurses being checklist coordinators, yet the nursing teams have taken on the responsibility of ensuring consistent use of the checklist [12]. To understand interactions among the personnel, and hence the context or setting in which people address a problem or an issue, there is a need to conceptualize the behavior and work role perceptions of the nurses, conducted with a qualitative study [14]. By using a grounded theory approach we therefore aimed to explore the nurse anesthetists and operating room nurses’ challenges and strategies used when utilizing the WHO’s checklist.

## Methods

Grounded theory is a qualitative, systematic approach used to explore processes in the context of situated interaction, with an embedded focus on human action and interactions, and involves the concurrent collection and analysis of data to formulate theories that are grounded in the world of the participants [15-17]. The intent of this research method is to move beyond description, and to generate or discover a theory that explains the situated actions and interactions as they experience, engage with, and manage the phenomenon of study. This is done by focusing on the main concern or problem that the individuals’ behavior is designed to resolve [15-17]. The goal of grounded theory is thus to discover this main concern, and hence the social processes that explain how people continually resolve it. The main concern or problem must be discovered from the data.

### Participants and setting

The study was conducted using a combination of methods including observation, a single interview with an OR nurse and focus group interviews of operating room nurses and nurse anesthetists. With an inductive perspective, the focus group interview guide was developed based on the observation of the nurses’ utilization of the WHO’s Safe Surgical Checklist. The study was carried out at a tertiary hospital in the Western part of Norway. The hospital has 1,100 beds, 12,000 employees and is a referral hospital for 600,000 inhabitants with a surgical volume of 25,000 operations annually. The Safe Surgical Checklist had been routinely used for nine months. The venue of the observation was at the hospital’s central operating theatre in a neurosurgical operating room. The operating teams consisted of two surgeons, three operating room nurses, one anesthesiologist and two nurse anesthetists. The Safe Surgical Checklist utilization was the object of the observation. The operating team and patients were sampled in permission with the unit management. Nurse anesthetists and OR nurses were recruited into focus group interviews in collaboration with the unit management. The first focus group interview included three nurse anesthetists and the second group interview included three OR nurses. Based on the findings in the analysis of the first two interviews, we conducted a single interview with one OR nurse to validate the process of the emerging theory [15-17]. The composition of the two last focus groups was designed purposefully, consisting equally of both two nurse anesthetists and two OR nurses, due to the emerging conceptualization. The inclusion criteria was having experience with the WHO’s checklist performance over a 9-12 month period. Out of 14 nurses (n = 14) two were male, and the range of work practice experience in the OR varied from 1-29 years.

### Data collection

The observation of the checklist in use took place in the OR where the nurses performed their daily work, and covered one operation, one team and their use of the checklist in the OR for one day. A total of four focus group interviews were combined with one single interview. In grounded theory, collecting data is not envisioned as a single, unidirectional line, but its process is guided by the developing grounded theory [15-17]. Observational data was noted during and immediately after the observed operation. The notes were subsequently analyzed and were inherently the basis for development of the focus group interview guide Additional file 1, and validation of interviewer’s role.

The observation and the interviews were conducted over a time period of four months.

Each of the four focus group interviews lasted 45-60 minutes and the single interview lasted 40 minutes. A nurse, trained as a moderator, assisted in two of the four focus group interviews. To initiate free discussion open ended questions were used, and the interviews were conducted in hospital localities free of disturbance [14]. The two first focus group interviews were carried out using the first version of the interview guide, whereas an edited interview guide was used to conduct the last three interviews. All interviews had the same opening question: “Can you tell me what it has been like, using the WHO’s “Safe Surgery Checklist?” While the first interview guide focused on barriers and motivators when using the checklists in general, the second interview guide focused on the challenges reflecting conditions and adaptive processes towards specific parts of the checklist. All interviews were recorded and transcribed verbatim. In accordance to the grounded theory, each transcript was analyzed before the next interview. Sampling was controlled by the emerging theory as in theoretical sampling, according to Glaser, 1978 [16].

### Data analysis

The transcripts were analyzed using the constant comparative method: each interview was analyzed and compared to the previous interview combined with written textual notes from the checklist observation in a continuous process [15-17]. In accordance with grounded theory methodology, an open coding was performed manually line-by-line, by the first author, constantly focusing on the incidents: the meaning, action, and interaction of “what is actually going on in the area studied”. The nurses’ main concern was identified after the observation in the OR and the performance of two focus group interviews and one single interview. The last two focus group sessions were held to ensure variety in the data-material, and to enrich the emerging codes and hypotheses. Saturation in data was achieved after analyzing the fourth focus group interview, and the study progressed to identify patterns of behavior by which the nurses resolved their concern. Examples of an open coding from data are presented in Additional file 2: Table S1. The codes were subsequently grouped into broader, tractable categories, and further into more extensive, universal categories, thereby translating the descriptive concepts until theoretical saturation was obtained [15-17]. During the whole process of analysis; memos, theoretical ideas about codes, categories and their relationships were written and used in the analysis. When the core category was identified, it was finally compared with the literature in the field according to Glaser, 1992, to see if the findings were supported [17].

### Trustworthiness

To ensure trustworthiness of the study and its findings, verbatim transcription, constant comparison, and persistent and prolonged engagement with the data were used [14]. Peer debriefing sessions as described in Creswell, 2007, were also held to provide an external check of the research process [14]. This involved health care professionals from both the hospital and the local University College. Assessing the quality of grounded theories requires analyzing the criteria of fit, work, relevance and modifiability [15-17]. That is, the theory *fits* the data, and works to explain the variation within the data set. “Discovered” like this, a grounded theory will fit empirical situations. The notion of *work* means that theories should provide predictions, explanations and interpretations of what is going on in the area under study. *Relevance* implies that theories should be relevant to action in the area it purported to explain, focusing on the emerged core problems and processes. The notion of *modifiability* means that a grounded theory might go through changes when new data emerge, generating qualifications to the theory [15-17]. Our data came from Norway, from a hospital in which the checklist had been in use for a 9-12 month period, but without a systematic evaluation of its use. Studying the nurses appointed as checklist coordinators would therefore probably provide valuable predictions and explanations of the checklist usage.

### Ethics

This study was not undertaken by Norwegian law according to the Committee for Medical Research Ethics of the Western Health Region of Norway. Approval for this study was given by hospital management and the data privacy Ombudsman at the tertiary teaching hospital involved. All respondents gave their informed consent to participate in the interviews. The patients and personnel present at the OR during the utilization of the checklist also gave informed consent.

## Results

The participants’ main concern is seen as both the cause and motivation for their actions [15-17]. It was therefore essential to capture this. The nurses’ main concern was identified as “how to obtain professional and social acceptance within the team”, which seemed to be existentially contingent in their pre-existing environmental context in addition to performing the checklist. Consequences of the actions performed by the nurses, consciously or not, to gain recognition from the team, were however characterized by how they related to both their task work and the teamwork in the OR, including the social interaction among the surgical team members caused by the checklist. The emergent grounded theory of “adjusting team involvement” explained how they resolved their challenge. Adjusting team involvement consisted of three strategies: distancing, moderating, and engaging social and professional team involvement. Each strategy had corresponding conditions and consequences, which determined checklist compliance, and how the checklist was used. The strategies also varied according to the different phases of the checklist, and the team compositions. The nurses directed their strategies according to their professional role, relations with the team members, and to their activities in the OR. The three different strategies are presented in Additional file 3: Table S2 with their corresponding conditions and consequences.

### Distancing team involvement

Conditions of this strategy were mainly characterized by uncertainty and lack of consensus as to when, where, in which situations, and in what types of surgery the checklist was to be used. This caused frustration among the nurses, and many of them expressed a need for administrative guidelines, and management commitment regarding the implementation of the checklist. They also seemed to struggle in preparing for the scheduled procedures with limited time available, thus regarding the checklist as another task they “had to do”. A considerable resistance in the team towards checklist use was also experienced by the nurses. This resistance was expressed verbally in an active ridiculing manner, and non-verbally in a more ignorant manner.

*“If the surgeon is in a bad mood when we initiate the use of the checklist the first time, then I won’t be bothered with it anymore, it affects me as well, and it is so incredible fatiguing. I think to myself,” well, well,” and I leave it* [the checklist] *aside, and I simply let it be!”*

Another example described was a surgeon who initiated the surgical procedure without getting involved in and simply ignoring the use of the checklist.

“Well, if this is how it is going to be during the first scheduled surgery, I’m telling you quite honestly that the checklist ”will be forgotten” in the next scheduled surgery. I will not be bothered! It creates dissatisfaction, and an unpleasant mood in the OR!”

Under the influence of these barriers, in order to obtain professional and social acceptance within the team, the nurses strategically tried to reduce the negative atmosphere in the OR. This was done by primarily avoiding drawing negative attention towards themselves by becoming “invisible”. Strategies used were silent communication, strategically positioning themselves in the OR to avoid disturbances. As a result, the checklist was initiated in an imprecisely vague manner, thus making it difficult to follow. Instead of committing themselves to the team, they awaited other team members’ social or professional initiative. Temporarily, they chose to prioritize and perform their professional task work. Uncertainty due to lack of administrative guidelines and limited time available also caused a random use of the checklist. This was prominent during the presence of patients awake in the OR, especially before administrating anesthesia, but also when performing the rest of the checklist when the patient received local anesthesia. In these situations, the nurses were worried that the patients might become anxious when embedded risks of anesthesia were expressed aloud by the team. The nurses also had concerns that patients who were nervous or needed close attention might feel left alone while the health care personnel concentrated on the checklist. However, the limited usage of the “Sign In” part of the checklist seemed more as an oversight than an intentional action. This was due to the lack of consensual guidelines, as expressed by one of the nurses:

*“…rotation at the ward, being away from it* [the checklist] *for such a long time, it’s something we don’t do all the time, and we simply forget. It is not a routine yet, not at all, if you ask me!”*

A limited team involvement was the consequences of the nurses’ distancing strategies used to obtain professional and social acceptance within the team, under the influence of the conditions described when using the checklist.

### Moderating team involvement

Conditions of this strategy were mainly pragmatic and were characterized by concurrent tasks, differences in the nurses’ daily work pattern causing different priorities, yet a willingness to get involved in the team. The nurses seemed to have several competing duties to perform; preparing the surgical equipment, preparing the operating table, preparing for anesthesia; preparing medications and IV fluids, getting the patient to the OR on time, and getting the patient prepared for anesthesia. The nurses were also very conscientious as to their professional duties, and to what extent the checklist might be improved to ease their daily work tasks. They suggested several issues to be added to the checklist, although they were specific about not making the list too long. Suggestions made were questions about infectious diseases and implants (such as pacemakers), which were likely to require specific preparations. A pre-induction checklist [10] was already in use on the premises, which included some of the questions in the WHO’s checklist. The pre-induction checklist was carried out prior to the WHO’s checklist, and the nurse anesthetists therefore suggested to combine the two checklists.

In order to obtain social and professional acceptance from the team the nurses sought attention by approaching the team by exchanging information considering the patient and the scheduled procedure. Strategies used were questioning specific issues like administration of antibiotics or other medications, or the use of certain instruments if the written information was unclear. The nurses were clear about the potential of the checklist as a reminder to ensuring that existing routines and guidelines were followed when planning for a surgery, in addition to illuminate lack of such routines. This was evident with the following example of drug administration:

*“…you have to ask for it,* [type of medication] *otherwise it won’t be given! It doesn’t automatically follow the patient, so if I didn’t know any better, it’s not guaranteed that the patient would have received the intended dosage!”*

A rationalized approach was also used by the nurse anesthetists and OR nurses individually during the surgery. This was done to secure information concerning their specific professional needs even though this implied that information beneficial to the others was not said aloud. The timing aspect was also of importance when moderating their team involvement. This was due to the daily OR logistic.

“…the most useful information on the checklist is the length of the surgery. If the operation is scheduled for two hours, and the surgeon announces that the operation will endure for four hours, then I have received useful information! Or if he announces the opposite, this will take only one hour. Then I have gained information from the checklist that I wouldn’t have received otherwise!”

It was obvious that the nurses considered the checklist as most useful when its use provided them with information which they wouldn’t have received otherwise. Thus, in order to obtain social and professional acceptance from the team, the nurses rationalized their team involvement according to their practical and-or professional needs. This was done mainly by selecting when and to whom they got involved with in the multidisciplinary team even though this strategy didn’t involve the entire team.

“…when the surgeon finishes the procedure, we always speak up loud: the number of sponges and instrument are OK! We state this very clearly, to the surgeon… if the nurse anesthetist doesn’t listen, it doesn’t matter, and it has been controlled many times before- two times verbally, and two times electronically”

Their strategies were also related to challenging the barriers in the OR, rather than avoiding them. A selected team involvement was thus the consequence of the nurses’ moderating strategies used to obtain professional and social acceptance within the team, under the influence of the conditions described when using the checklist.

### Engaging team involvement

The dominant condition in this strategy was the nurses’ experience of a positive and respectful attitude towards one another within the surgical team, including the utilization of the checklist. A sense of respect and recognition among the team members seemed to already exist. This was seen through a positive response from the team when reviewing the different parts of the checklist, and the nurses reported this as a motivation for their team involvement. Less experienced nurses also emphasized the benefits they automatically gained from utilizing the checklist, compared to more experienced nurses.

“In my opinion, the checklist has been very useful for me as a novice! I get information about the scheduled surgery and its implications. Such a debriefing before we start has been of great importance!”

The nurses also perceived the checklist as being meaningful in itself, thus by using it they were contributing to quality improvements in the OR. Increasing the patient safety through participating in and coordinating the utilization of the checklist on a daily base, was considered an important work. The strategies used when engaging team involvement seemed therefore reliant to the nurses’ awareness of their own professional role; both as specialized trained nurses and as checklist coordinators. Their professional attitude also influenced the strategies they used. This involved taking leadership through requiring attention from the team. This was done verbally by speaking out loudly to the rest of the team, and nonverbally by strategically positioning themselves to get the team members attention.

*“Personally, I don’t think this has been a problem at all!* [utilization of the checklist] *I grab the checklist, and announce: TIME OUT! And I speak clearly and aloud, and I don’t give up until I get an answer! …If the surgeon ignores the checklist…we have to ignore them! We* [checklist coordinators] *have to take control!*”

Although resistance was detected, this was not a problem, as it was ignored. The nurses seemed to take control of the checklist utilization by coordinating and structuring the information exchange, speaking out clearly and aloud, from a central position in the OR.

If the surgeons were impatient during the utilization of the checklist, to draw attention toward themselves (and the checklist) the scalpel was sometimes hidden until the “Time-Out” had been completed. During the utilization of the checklist, the coordinator sometimes had to pursue the surgeon in order to get attention. This implied a prolonged “Time-Out” session. This was a concern to the nurses. Even though the checklist was completed, it was too time-consuming, implying a risk of losing the team members attention.

“If you’re doing the “Time-Out”, it should be a real time out! Everybody should stop and pay attention to the checklist. If everything is all right then we can go on!”

By engaging team involvement the nurses were positive about using the checklist and taking control of the coordination while making improvements suggestions. Thus, obtaining social and professional acceptance from the team was gained by utterly initiating their team involvement by taking responsibility for team actions included the checklist.

The consequence was thus an unlimited, interdisciplinary team involvement, with an embedded focus on prioritizing and using the checklist as intended.

## Discussion

Findings describe nurse anesthetists’ and OR nurses’ prerequisites to the utilization of WHO’s Safe Surgical Checklist including patterns of behaviors and consequences determining checklist compliance, and how the checklist was used. Three main patterns of behavior emerged correlating to the practical, social, and professional conditions involved in the OR; distancing, moderating, and engaging team involvement. However, these strategies must not be seen as phases in a progressive process, but more as patterns, reflecting the present sociocultural and practical work environment. This implies that the nurses might change their strategy during the day according to conditions such as team compositions, or competing task work. Different strategies might also occur simultaneously due to the nurses being present in the OR and their individual perceptions of the conditions involved. The nurses’ main concern in relation to the surgical team was however not linked directly to the utilization of the checklist involved, but as how to obtain social or professional acceptance within the team in general. The checklist seemed to be merely one occasion where that issue had arisen. In order to get involved in and within the surgical team, thus taking on the responsibility to perform the WHO’s checklist as intended, the nurses desired a degree of social or professional acceptance within the team. This was obtained by the three particular patterns of behaviors as mentioned, which we have conceptualized as “adjusting team involvement”. This adaptive strategy indicates that the checklist itself does not work as a tool to optimize communication patterns and collaborative work processes, and further enhancing patient safety as intended. However, through the utilization of the checklist key issues in team collaboration that were currently invisible to team members and seemed integrated as part of the culture, were made visible. In this section we highlight the safety relevance of adjusting team involvement.

### Task work and teamwork

A combination of elements related to both task work and teamwork are incorporated in the checklist. Whereas *task work* refers to behaviors that are related to task execution, such as the interaction with medical equipment- and instruments, *teamwork* is about how team members organize their joint actions [8]. Factors involved in teamwork include designating roles, determining the timing of activities, coordinating action, and managing interpersonal factors such as decision making and conflict resolution [18]. Thus, *team involvement* can be seen as the active or passive strategies which members of a team perform, unconsciously or not, in order to join in the team, either socially, or professionally. In our study the dominating barriers to intentional use of the checklist were lack of consensus guidelines, resistance within the team, uncertainty, and competing task work. As many of the issues addressed in the checklist seemed to be incorporated in pre-existing procedures performed by the nurses, when barriers were perceived, these work tasks were performed and accounted for regardless of the checklist. One example was checking the patient’s identification and use of medications prior to surgery. The conceptualized strategy of “selecting team contact” was used when the nurses made arrangements of information exchange within their own group, instead of involving themselves in the team, thus confirming this information aloud to all the team members by utilizing the checklist. Duplication of existing processes covering items in the checklist, clashing clinical priorities to performing “time out”, ambiguity about the response systems, lack of communication between the surgeon and the anesthetist, time consumption, general communication problems, hierarchical team culture and tribal affiliations of members, have also been described as barriers to intentional use in other studies [19,20]. Additionally, concerns about the legal implications of signing the checklist as being held accountable for errors, have also been expressed by nurses in the OR elsewhere. [19]. Furthermore, a poor use of the checklist has also shown a potential to deepen existing cultural divisions and further fray inter-professional dynamics [12], in addition to giving a false sense of security and compromising safety and teamwork [9]. In order to deal with and overcome logistical, structural and cultural barriers towards checklist utilization, the recognition of its utility, as described by Lingard and colleagues, [21] is of crucial importance. This model describes a causal pathway where team members can be seen to move from sharing information, to identifying a problem that threatens the safety of care, to decision- making to resolve that problem, and finally to follow-up actions to enact the new plan [21]. However, even though nurse anesthetists and OR nurses seemed to have a loyal attitude towards the checklist considering their task work, they adjusted their team-involvement according to practical, social and professional conditions in their work environment. This indicates that logistical and structural barriers are inferior to the cultural barriers. Thus, to improve safety culture in the OR, interventions should aim at minimizing the hierarchy and empower nursing staff, in addition to standardizing and structuring the practicalities concerning the utilization of the checklist. Such initiatives need to be carried out by the OR management.

### Safety culture in the OR

The working culture of an organization has been described as “analogous to the personality in the individual”, with the embedded complex pattern of beliefs, values, attitudes, norms and unspoken assumptions of all the people that behave and work together [22]. Even though the OR culture may be hard to define and measure, the culture may lead to different expressions. This can be measured by patterns of communication and team cooperation, such as utilizing the checklist. As seen in our study, adjusting team involvement was a response both to structural conditions such as lack of consensus guidelines, and cultural conditions such as communication difficulties. Furthermore, the behavioral patterns of adjusting team involvement were linked directly to collaborative processes.

Even though patterns of communication in the OR are known to be complex and socially motivated [23], the aim of the checklist is nevertheless to reinforce accepted safety practices and to foster better communication and teamwork between clinical disciplines. However, discrepancies in perception of teamwork are known to exist in the OR. Interdisciplinary diversity in teams contributes to complex interpersonal relations [24]. Nurses often describe good collaboration as having their input respected, whereas physicians often describe good collaboration as having nurses who anticipate their needs and follow instructions [25]. This correlates with the findings in our study where the nurses limited their team involvement by avoiding drawing attention towards themselves, when the coordinating of the checklist was met with resistance within the team. Even though issues concerning the patient safety were compiled into the checklist, deficiencies in responding to questions in the checklist were seen. This indicates that the real challenges to utilizing and to fully realizing the benefits of the checklist are cultural. As the core element of patient safety work implicates an understanding of situations which might lead to adverse events, it is important to realize that the implementation of the checklist will require an adaptive solution. Expecting that a straight forward technical solution will solve an adaptive, cultural problem is too simplistic. Therefore, the safety checklist will be of little value if disruptive attitudes and behaviors are not addressed [26]. In a safety perspective, root cause analysis have revealed that adverse events often are caused by failure to fully appreciate how one profession’s competent performance is dependent on the other’s [4], and that the perception of safety being about individual competence which persists as a standard and expectation in the healthcare field [21]. As seen in our study, the nurses downgraded the intentional utilization of the checklist by avoiding negative attention, awaiting team initiative, and selecting focus as to whom they got involved with in the team, and which questions they addressed in the checklist as part of adjusting their team involvement. A discrepancy in experiences of a team briefing has also been described by Lingard and colleagues [21]. Whereas the nurses reported an appreciation of the additional information provided them via the briefings, the surgeons perceived the provision of information negatively [21]. A lack of awareness of other professionals’ roles and need for information to meet the required expectations of the surgery, might explain this lack of team collaboration. A significant discrepancy between the surgical team members regarding their perception of communication, teamwork, and situation awareness, has also been found in a study by Wauben and colleagues [23]. Surgical team members’ constructions of other professions’ roles, values and motivations also seem to vary with those professions’ constructions of themselves [27,28]. All team members have a tendency to overrate their own understanding of colleagues’ role in the OR, and this was especially marked for the surgeons [28]. However, novices’ echoing such role simplification has implications for their professional identity formation, and might also influence their attitudes towards checklist use.

As found in our study, the nurses’ main challenge was how to obtain social and professional acceptance within the team. As the lack of such basic interpersonal skills and respect of different professional’s roles has been identified as causes for compromised communication [29], an understanding and respect of each other’s roles in multidisciplinary teams should be part of the curriculum for both nurse and medical students. Education as central to changing culture and increasing understanding among interdisciplinary team members has also been described by Gillespie and colleagues [24]. In a Cochrane review such an interprofessional education has produced positive outcomes in collaborative team behavior and reduction of clinical error rates for emergency department teams [30].

### Strengths and limitations of the study

Implementing the WHO’s checklist implies a change for the people involved. Grounded theory methodology developed by Glaser and Strauss, 1967 with an embedded focus on human action and interaction [15-17] is well suited for studies of peoples’ responses to change. Although all interviews were uni-disciplinary, reflecting exclusively the views of the nurse professionals involved, the combination of homogenous- and mixed groups, complemented each other. The quality of the data was thus considered rich enough to achieve variation, saturation, thus maintaining depth in the analysis. The interviewer’s interest in the checklist and implementation process was clear to the participants, and the interviewer also had pre-existing work relationship with some of the participants. This might have influenced the nurses’ responses which additionally might have been interpreted as being somewhat biased because of the lack of medical representation. However, in addition to the interviewer’s similar professional background, this facilitated the discussion.

Assessing the quality of grounded theory requires analyzing the criteria of fit, work, relevance and modifiability [15-17] and it is supportive of the theory of adjusting team involvement that emerged from our data. The theory also seems relevant to the strategies conducted by the nurses in the OR. As for the concept of modifiability, the theory of adjusting team involvement might well adapt to changes when new data emerge. This is yet to be seen, thus more research on the subject is needed. However, empirical data has shown that lack of awareness and motivation, as well as perceived external factors, is particularly important barriers to adopting and utilizing evidence based guidelines [31]. In addition, Mickan et al. have shown in a systematic review that leakage from guideline publication to utilization occurs among different specialties, across a range of recommendations, in different countries and healthcare systems [32]. The production and dissemination of evidence based clinical guidelines is not sufficient to ensure that research evidence gets into practice, as with the WHO’s Safe Surgery Checklist. As for the nurses involved in our study utilizing the checklist as intended seemed to be part of an existential challenge; obtaining social or professional acceptance within their team.

## Conclusion

Prerequisites for nurses’ patterns of behavior and work practice in compliance to the WHO’s Safe Surgical Checklist are described in this study. According to the WHO the checklist provides a tool for two purposes: enabling consistency in safety for patients, and introducing and maintaining a culture which values achieving it. However, our findings show that within a surgical team, obtaining social and professional acceptance within the team seems to be of crucial importance for nurses to involve themselves in the team and fully participate in the performance- and use of the checklist. Even though nurses seem to have a loyal attitude towards the WHO’s checklist regarding their task work, they adjusted their surgical team involvement according to practical, social and professional conditions in their work environment. This resulted in an incomplete use of the checklist and therefore a low compliance rate. Findings also emphasized the importance of management support when implementing WHO’s Safe Surgical Checklist, and a team–based approach to local adaptation of the checklists use. Building expectations of performance standards into work processes, when introducing the WHO’s “Safe Surgical Checklist”, might contribute to improve the culture. Further research should explore strategies to strengthen social and professional acceptance within the surgical team in order to improve team involvement.

## Competing interests

The authors declare that they have no competing interests.

## Authors’ contributions

HVW and EH were responsible for the study conception and design. HVW performed the data collection. HVW and EH were responsible for the data analyses. The analysis and the emergent themes were by supported ASH and ES. HVW was responsible for the drafting of the manuscript. ASH, ES and EH made a substantive contribution to revising the paper. All authors have read and approved the final manuscript.

## Pre-publication history

The pre-publication history for this paper can be accessed here:

http://www.biomedcentral.com/1472-6955/11/16/prepub

## Supplementary Material

Additional file 1 Semi-structured interview guide I. Click here for file

Additional file 2 **Table S1. **Example of open coding from data. Click here for file

Additional file 3 **Table S2. **Adjusting team involvement. Click here for file
