# Supplementary material for: Adjusting team involvement: a grounded theory study of challenges in utilizing a surgical safety checklist as experienced by nurses in the operating room
Source: BMC Nurs. 2012 Sep 7;11:16. doi: 10.1186/1472-6955-11-16 (PMC3499446; doi:10.1186/1472-6955-11-16)
Supplement: Additional file 1 — Semi-structured interview guide I. [file 1472-6955-11-16-S1.doc]

**Semi- structured interview guide I:**

**Opening question:**

- Can you describe how it has been like utilizing the”Safe Surgical Checklist”?

**Theme question:**

- Can you describe a situation where it has been a useful, or a positive experience utilizing the checklist?
- Can you describe a situation where it has been difficult utilizing the checklist?
- How do you feel that the checklist influences the surgical team?
- How do you think the checklist affects your professional duties in the surgical team?
- What do you think will promote the checklist utilization? Why?
- What do you think will hinder the checklist utilization? Why?

**Closure questions:**

- Do you have anything you would like to add?
- What has it been like to be part of this focus group?
