# Supplementary material for: Adjusting team involvement: a grounded theory study of challenges in utilizing a surgical safety checklist as experienced by nurses in the operating room
Source: BMC Nurs. 2012 Sep 7;11:16. doi: 10.1186/1472-6955-11-16 (PMC3499446; doi:10.1186/1472-6955-11-16)
Supplement: Additional file 2 — Table S1. Example of open coding from data. [file 1472-6955-11-16-S2.doc]

**Table S1: Example of open coding from data**

| Indicators in data | Incidents in data | Property of category | Categories of substantial coding |
| --- | --- | --- | --- |
| ”The surgeon, some surgeons are maybe less motivated to perform the checklist?.. and they think that we don’t need to spend time doing this? But, we might have spent more time doing this to begin with!”  “This is exactly what I find troublesome! The surgeons are disrespectful! Very often!  When the OR nurse tries to perform the checklist, they turn their back on the team, and mumble behind their surgical masks… they are not taking this very seriously?”  “In my opinion, many of them do this, [incidents in performing the checklist] which is obviously a weakness with the checklist..” | Perceiving lack of motivation among others  Experiencing challenges  Trying to perform the checklist  Observing non-verbal resistance  Requesting surgeons commitment  Requesting seriousness  Clarifying checklist performance weaknesses | Miss common commitment  The checklist is not perceived as an integrated routine  Acknowledge checklist resistance within the work-environment | Require practical, consensual guidelines  for checklist use  Require management initiative |
