# Supplementary material for: Adjusting team involvement: a grounded theory study of challenges in utilizing a surgical safety checklist as experienced by nurses in the operating room
Source: BMC Nurs. 2012 Sep 7;11:16. doi: 10.1186/1472-6955-11-16 (PMC3499446; doi:10.1186/1472-6955-11-16)
Supplement: Additional file 3 — Table S2. Adjusting team involvement. [file 1472-6955-11-16-S3.doc]

**Table S2**: Adjusting team involvement

| **Strategies** | **Distancing** | **Moderating** | **Engaging** |
| --- | --- | --- | --- |
| **Conditions**  **Adaptive**  **Process** | - Lack of consensus guidelines - Resistance in the team towards checklist use - Uncertainty | - Competing task work - Professional consciousness | - Existing recognition among team members - Positive team response on checklist use |
| **Individually** | - Avoiding attention | - Seeking attention | - Demanding attention |
| **Socially** | - Selecting team contact | - Rationalizing initiative | - Coordinating initiative |
| **Professionally** | - Limiting interdisciplinary team involvement | - Selecting interdisciplinary team involvement | - Initiating interdisciplinary team involvement |
| **Consequence** | - **Limited use of the checklist** | - **Selective use of the checklist** | - **Unlimited use of the checklist** |
